# Gastric metastasis of renal cell carcinoma: features, mechanisms, and insights from existing literature

**DOI:** 10.3389/fcell.2025.1656858

**Published:** 2025-10-15

**Authors:** Xiaoqian Wang, Luling Wei, Runchang Liu, Xuezhe Wang, Xinchi Luan, Xiaoxuan Li, Haoran An, Ruizhe Zhao, Yue Qiu

**Affiliations:** ^1^ Department of Nephrology, The Affiliated Hospital of Qingdao University, Qingdao, China; ^2^ School of Basic Medicine, Qingdao University, Qingdao, Shandong, China; ^3^ Department of Cardiology, The Affiliated Hospital of Qingdao University, Qingdao, China; ^4^ Department of Oncology, Key Laboratory of Cancer Molecular and Translational Research, The Affiliated Hospital of Qingdao University, Qingdao, Shandong, China; ^5^ Department of Urology, Qilu Hospital of Shandong University, Qingdao, China

**Keywords:** epithelial-mesenchymal transition, extracellular matrix remodeling, gastric microenvironment, metastasis, renal cell carcinoma

## Abstract

Renal cell carcinoma (RCC), a malignancy characterized by an increasing global incidence, exhibits a tendency for metastatic dissemination. However, gastric metastases, often identified in multicenter case series with an incidence of 0.2%–0.8%, typically present years after nephrectomy (median interval ∼6.7 years) and are associated with a poor prognosis (5-year OS ∼21% in historical cohorts). Gastric metastases typically present years after nephrectomy as either isolated or polymetastatic lesions, often accompanied by severe upper gastrointestinal symptoms and presenting significant clinical challenges. Mechanistically, the progression of metastasis is driven by dysregulated signaling pathways, including PI3K/AKT, Ras/MAPK, and Wnt/β-catenin, which facilitate epithelial-mesenchymal transition (EMT), extracellular matrix (ECM) remodeling, and angiogenesis. The gastric microenvironment further contributes to tumor adaptation through metabolic stress, immune evasion, and exosome-mediated intercellular communication. Clinically, oligometastatic disease may benefit from surgical resection in combination with immunotherapy, whereas polymetastatic cases necessitate systemic therapies such as tyrosine kinase inhibitors and immune checkpoint blockers, albeit with limited efficacy. Emerging multi-omics approaches and single-cell sequencing technologies hold promise for elucidating organ-specific tropism and refining personalized treatment strategies. This review highlights the critical need to integrate mechanistic insights with innovative therapeutic interventions to improve outcomes for patients with gastric metastasis of RCC.

## 1 Introduction

Renal cell cancer (RCC) is the twelfth most common cancer worldwide, whose incidence rate is increasing over these years (Siegel et al., 2022; Siegel et al., 2023; Siegel et al., 2024; Zhang L. et al., 2024; Rose and Kim, 2024). In 2024, it was estimated to occur in 81610 patients in the United States, resulting in 14390 deaths with a mortality rate of up to 17%. Among them, RCC accounts for 80%–85% of kidney cancer, which is prone to occur at the lateral upper pole of the kidney (Petejova and Martinek, 2016). RCC rises from renal tubular epithelial cells. And there are five types of RCC, including 75%–85% clear cell carcinoma, 12%–14% chromophilic cell carcinoma, 4%–6% chromophoric cell carcinoma, 2%–4% oncocytic carcinoma, and 1% collecting duct carcinoma (Rini et al., 2009). About seven or eight out of ten patients still face a poor outlook, and three out of ten patients later learn the cancer has spread to lymph nodes, lungs, liver, contralateral kidney, adrenal gland, brain, bones, stomach and so on (Gulati et al., 2024; Bahadoram et al., 2022). The overall 5-year survival rate is about 77%, with a late-stage survival rate of only 15%. Because RCC can be deadly and its causes are not fully understood, its mechanism is vital to identify, and more research is needed to find better treatments.

Gastric metastasis from any primary tumor is uncommon, and involvement by RCC is especially rare, being reported in only 0.2% of the literature (Yan et al., 2024). The most common primary malignant tumor of gastric metastasis is breast cancer (95/340, 27.9%), followed by lung cancer (81/340, 23.8%) and esophageal cancer (65/340, 19.1%) (Namikawa and Hanazaki, 2014). There are two types of gastric metastasis in RCC, one is to metastasize to various organs throughout the body, including the stomach, for example, to simultaneously metastasize to organs such as the stomach, lungs, and liver; Another type is isolated single gastric metastasis that only metastasizes to the stomach, which is relatively rare (Kim et al., 2012; Degerli et al., 2022). Compared with breast or lung cancer, RCC displays a protracted metastatic timeline. While the average interval from primary diagnosis to gastric involvement is 1.3 years (Degerli et al., 2022), gastric metastasis may emerge after an average latency of 6.7 years, rendering this complication both insidious and diagnostically elusive (Namikawa and Hanazaki, 2014; Pollheimer et al., 2008). And its symptoms are more severe. The initial symptoms of RCC gastric metastasis patients are upper gastrointestinal bleeding, gastroesophageal reflux, anemia, black stool, and upper abdominal pain, or may be gradual (Namikawa and Hanazaki, 2014). Endoscopically, lesions are often submucosal masses, deep ulcers or, rarely, fundic-gland polyps (Degerli et al., 2022). Generally, the outcome of patients with RCC and gastric metastasis is poor. Some patients die within a few weeks (Kim et al., 2012). Most patients do not experience tumor recurrence within a median of 12 months (range 6–18 months) after undergoing metastatic tumor resection surgery (Kim et al., 2012).

As the incidence of RCC continues to rise and research remains limited (Li et al., 2025; Zheng Z. et al., 2024), this review summarizes current knowledge on gastric metastasis and its underlying mechanisms, aiming to enable earlier detection and improved patient outcomes.

## 2 Search strategy and selection criteria

### 2.1 Search strategy

With the assistance of an information specialist, a search strategy was developed and refined using Boolean operators. The following search terms were used in the PubMed database: (“Renal Cell Carcinoma” OR RCC) AND (Metastasis OR metastatic) AND (Stomach OR Gastric). The search process and reporting were conducted in accordance with the PRISMA (Preferred Reporting Items for Systematic Reviews and Meta-Analyses) guidelines. The review protocol was not pre-registered. The initial search yielded a total of 365 articles. After removing duplicates, records flagged by automation tools as non-compliant, and excluding others for various reasons, 362 articles were screened. Of these, 139 articles were excluded for not discussing stomach metastases separately, and 34 were non-English articles. Ultimately, 189 articles were identified for full-text retrieval, with seven unable to be obtained. Following further eligibility assessment, 182 reports were evaluated, with 2 excluded due to severe data incompleteness, resulting in a total of 180 studies included for analysis.

### 2.2 Study inclusion and exclusion criteria

**FIGURE 1:**
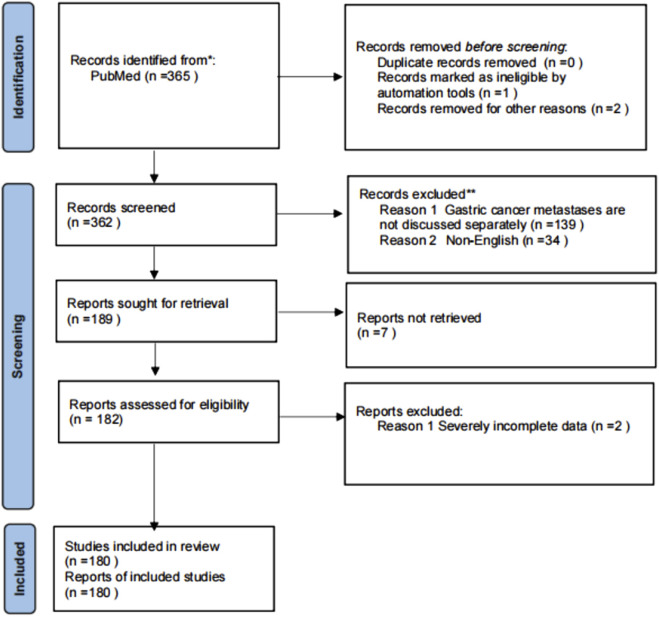
Flow chart of included studies.

This study systematically searched and included relevant original studies, conference abstracts, and case reports published between 1 January 2000, and 31 December 2025. The included literature was required to be based on human renal cancer tissue specimens and include immunohistochemical analysis. The exclusion criteria were as follows: (1) studies involving only cell lines or *in vitro* models; (2) studies focusing primarily on hereditary renal cancer syndromes; (3) studies that only analyzed recurrent renal cell carcinoma or advanced patients without independent analysis of metastatic sites; (4) studies restricted to subjects with renal failure, dialysis, or kidney transplant recipients; (5) studies with incomplete data or where key information could not be extracted. Criteria were specified *a priori*. The PRISMA flowchart is in Figure 1.

### 2.3 Study selection

Two independent reviewers (Luling Wei, Runchang Liu) screened titles/abstracts. Discrepancies were resolved by a third reviewer (Xiaoqian Wang).

## 3 The overview of RCC

### 3.1 Classification and pathological features of RCC

As mentioned earlier, RCC is broadly classified into clear cell carcinoma, papillary RCC, chromophoric carcinoma, oncocytic carcinoma and collecting duct carcinoma (Xu et al., 2021; Vanderbruggen et al., 2022; B et al., 2024; Rizzo et al., 2023). Clear cell carcinoma is the most common pathological subtype of RCC, accounting for approximately 60%–85% of RCC (Xu et al., 2021). Its hallmark is the abundant accumulation of lipid droplets within tumor-cell cytoplasm, imparting a clear or pale-yellow appearance under light microscopy (Goswami et al., 2024). Papillary RCC, also termed as chromophilic cell carcinoma or Leber’s RCC, comprises 7%–14% of tumors and is composed of basophilic or eosinophilic cells arranged in papillary or tubular architectures (Linehan et al., 2016). Oncocytic carcinoma is characterized by large volume, presence of solid cystic components, bleeding, and necrosis, accounting for approximately 2%–4% of RCCs. The pathological feature of chromophobe cell carcinoma is that tumor cells are arranged in a papillary or tubular structure. And its incidence rate accounts for 12%–14% of RCC. Renal collecting duct cancer, arising from the epithelium of Bellini ducts, is rare (0.4%–2%) and is characterised by aggressive growth, early renal-vein invasion and rapid dissemination to regional lymph nodes, bone, lung and liver (B et al., 2024; Deng et al., 2023). The clinical manifestations of patients often include back pain, gross hematuria, and palpable abdominal masses (Hsieh et al., 2017).

### 3.2 Clinical presentation and diagnosis of RCC

Many patients with RCC only experience symptoms in the late stages, such as bone pain, worsening physical condition, or persistent coughing (Ljungberg et al., 2022). The typical triad of lower back pain, gross hematuria, and palpable abdominal masses is now rare and mostly associated with late-stage diseases and subtypes associated with poor prognosis (Ljungberg et al., 2019). Most tumors are detected incidentally on imaging. Paraneoplastic features such as hypercalcemia, pyrexia of unknown origin or polycythemia may also be present. Patients suspected of RCC should immediately undergo laboratory tests for serum creatinine, hemoglobin, white blood cell and platelet counts, lymphocyte to neutrophil ratio, lactate dehydrogenase, C-reactive protein, and serum corrected calcium, and other relevant symptom-based tests (Escudier et al., 2016). Imaging examinations play an important role in the staging, preoperative planning, and follow-up of RCC. The main imaging methods for diagnosing RCC include US, computed tomography, and magnetic resonance imaging. CT examination can detect RCC with small lesion diameter, manifested as abnormal masses on the kidney accompanied by extensive tumor infiltration into the renal parenchyma, as well as perirenal soft tissue retention (Sankineni et al., 2016). Ultrasound (US) can be used for the initial evaluation of indeterminate renal masses. It is a valuable and non-invasive first-line imaging tool, but its role is primarily for screening and triage rather than definitive diagnosis. RCC on ultrasound may appear as a solid mass that is hypoechoic or isoechoic relative to the renal parenchyma, or it may present as a complex cystic mass with internal septations, wall thickening, or enhancing solid components (nodules). These features typically correspond to Bosniak category IIF, III, or IV lesions and often necessitate further specialized renal mass protocol CT or MRI for more precise staging and definitive diagnosis (Sankineni et al., 2016; Krishna et al., 2020). Compared to the above two methods, MRI provides superior soft-tissue contrast, clearly delineating septa, solid components and intra-tumoral hemorrhage, and is reserved for problem-solving or surveillance (Krishna et al., 2020). However, in the process of rapid improvement, CT remains the preferred method for diagnosing RCC, while US has become the most commonly used technique due to its convenience, and magnetic resonance imaging has become an auxiliary way to monitor patients. Additionally, the diagnosis of RCC can also be achieved through renal tumor biopsy (Ljungberg et al., 2019). Coarse needle biopsy is superior to fine needle aspiration and is avoided in purely cystic lesions due to its low diagnostic rate. Histopathology obtained from biopsy or nephrectomy specimens supplies key prognostic data, including nuclear grading, sarcomatoid features, vascular infiltration, tumor necrosis, collection system and perirenal fat infiltration, as well as evaluation of LN status (Ljungberg et al., 2022). In addition, histopathological diagnosis of malignant tumors can be obtained through renal core biopsy or partial or radical nephrectomy specimens (Cohen et al., 2005). A confirmatory biopsy is therefore recommended before ablation in non-surgical candidates or before starting systemic therapy in metastatic disease.

For local RCC, surgery remains only curative treatment method, which can be divided into radical nephrectomy (RN) and partial nephrectomy (PN). Tumors with a diameter of less than 7 cm are preferentially treated with PN; whereas kidney tumors larger than 7 cm are managed by open RN as the standard approach. In metastatic RCC, resection of the metastatic lesion is still the appropriate local treatment method for most metastatic sites by default. In addition, drug-eluting bead transarterial chemoembolization (DEB-TACE), a technique that allows drugs to slowly release into tumor tissue after arterial embolization, has demonstrated excellent efficacy in RCC patients with hepatic and retroperitoneal lymph node metastasis (Qiu et al., 2024).

## 4 Gastric metastasis of RCC

RCC is recognized as a tumor with a pronounced propensity for haematogenous spread (Carneiro et al., 2024). About 20% of RCC patients develop metastatic disease at diagnosis, and approximately 50% of patients who undergo nephrectomy will develop metastasis (Kim et al., 2012). Gastric metastasis of RCC is a relatively rare phenomenon, with population-based studies and single-center retrospective case series, as well as multi-center retrospective case series, estimating its incidence to be between 0.5% and 0.8% (Carneiro et al., 2024; Yamashita et al., 2024; Namikawa et al., 2012). The median age of gastric metastasis in RCC is 67 years old, and the most common metastatic lesions are in the gastric corpuscle, followed by the gastric fundus and antrum, ranging in size from 0.5 to 10cm, usually found in advanced RCC patients (Degerli et al., 2022). It is usually found in patients with advanced RCC that the incidence rate of men is higher than that of women (Yamashita et al., 2024). The incidence of gastric metastasis is 0.65% (Yamashita et al., 2024), but isolated gastric metastasis rarely occurs. Once it occurs, it is often accompanied by metastasis of other organs, such as lung, bone, liver and brain (Kim et al., 2012). Metastatic tumors may appear as gastric masses on CT or endoscopy, and their clinical manifestations may be similar to gastric cancer (Bernshteyn et al., 2020). Upper-abdominal discomfort, poor appetite and dyspepsia are the symptoms most frequently reported by affected patients.

While liver and brain metastases are widely recognized as harbingers of dismal prognosis in advanced malignancies (median OS < 18 months), the clinical significance of gastric metastasis in RCC deserves particular attention (Carneiro et al., 2024; Zhuang et al., 2019). Notably, gastric metastasis rarely occurs in isolation - over 70% of cases present with synchronous metastases to high-risk organs like lungs, bones, liver, or brain, creating a compounded negative impact on survival (Kim et al., 2012; Carneiro et al., 2024). The median survival for gastric metastasis (19 months; range, 1–36) appears marginally better than that for liver or brain metastases (<18 months) (Zhuang et al., 2019). But this difference becomes less meaningful when considering that 1) gastric metastasis typically coexist with these high-risk metastases, and 2) the 5-year OS rate plummets to 21% (Carneiro et al., 2024; Saifi et al., 2023). This survival pattern contrasts sharply with metastases to endocrine organs (median OS > 27 months for pancreatic/thyroid/adrenal metastases) and other gastrointestinal sites (median OS > 37 months for duodenal/small bowel metastases). The particularly aggressive nature of gastric involvement is further evidenced by its association with rapid clinical deterioration - tumor progression frequently causes life-threatening complications like hemorrhagic shock, with some patients succumbing within weeks of diagnosis (Mori et al., 2024). These data collectively suggest that gastric metastasis, while numerically less common than liver or brain metastases, signals aggressive biology and still worsens survival through bleeding, perforation, or obstruction. The 6.5-year interval from nephrectomy to gastric metastasis paradoxically reflects this dual role, while occurring later than typical visceral metastases, its emergence signals transition to a terminal disease phase characterized by multi-organ failure and catastrophic clinical events (Carneiro et al., 2024; Saifi et al., 2023). Once stomach lesions appear, cure is rarely possible. Therefore, understanding and comprehending gastric metastasis is of great significance for exploring effective treatment methods in clinical practice. It is important to note that the aforementioned estimates regarding incidence, interval to metastasis, and survival outcomes primarily originate from multi-center and single-center retrospective case series studies spanning several decades. These studies cover heterogeneous patient populations receiving treatment in different therapeutic eras, introducing significant temporal heterogeneity. Additionally, due to the rarity of gastric metastases, sample sizes are typically limited, and precise confidence intervals are often unavailable. Therefore, these data should be interpreted with caution. Nonetheless, these retrospective cohorts provide valuable real-world insights into the natural history and clinical behavior of this uncommon pattern of metastasis, forming a fundamental understanding for guiding further mechanistic and therapeutic research.

We summarized the clinical characteristics of 18 reported cases of gastric metastasis from renal cell carcinoma (RCC) between 2007 and 2024 (see Supplementary Table S1 for details). Analysis of these cases revealed significant differences between oligometastasis and polymetastasis in terms of latency period, treatment response, and survival outcomes, with a detailed discussion provided in the “Treatment” section of Chapter.

## 5 Effects of gastric microenvironment on RCC metastasis

The gastric microenvironment critically shapes the behaviour of RCC after seeding to the stomach (Wang Y. et al., 2020). It has unique organizational characteristics, including a complex cell network composed of epithelial cells, stromal cells (such as fibroblasts and immune cells), and gastric glandular stem cells, as well as an extracellular matrix (ECM) rich in collagen and fibronectin and an acidic environment with low pH 1.5–3.5 (Zhang et al., 2019). These features force metastatic cells to undergo gene-expression and metabolic reprogramming: acid-tolerance genes (e.g., proton-pump subunits) are upregulated, glycolysis and glutaminolysis are intensified and nutrients are actively scavenged (Shang et al., 2024; Kumagai et al., 2022). And then, the dynamic remodeling of the gastric ECM is a crucial step in cancer cell invasion (Wang X. et al., 2022). Matrix-metalloproteinases (MMPs) secreted by tumour cells degrade the basement membrane, while recruited fibroblasts deposit a growth-factor-rich stroma that supports proliferation (Zhang et al., 2019; Wang X. et al., 2022). Concurrently, Tumor associated macrophages (TAMs) and other immune cells are skewed toward an immunosuppressive phenotype; PD-L1 over-expressed by tumor cells further dampens cytotoxic T-cell activity (Li et al., 2023; Li et al., 2024). Epithelial-mesenchymal transition (EMT) driven by gastric chemokines such as CXCL12, equips cells with enhanced motility and enables deep tissue infiltration facilitated by ECM remodeling (Qin et al., 2021). These adaptive changes interact with the microenvironment, forming a positive feedback loop that accelerates tumor progression (Piao et al., 2022). For example, the growth factors released by ECM degradation (such as TGF-β) can simultaneously promote EMT and immune suppression, while metabolites (such as lactate) further acidify the local environment, forming a vicious cycle (Xia et al., 2022). Thus, physical barriers, metabolic stress and immune modulation jointly dictate the fate of gastric-metastatic RCC cells.

## 6 Mechanism of RCC metastasis

**FIGURE 2:**
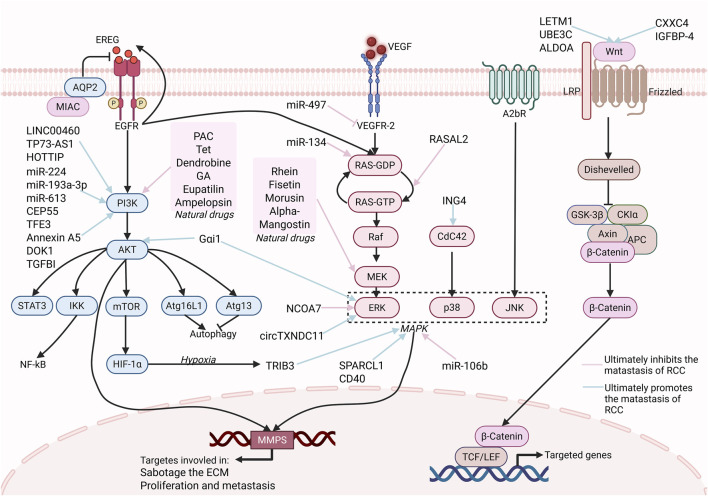
Major Signaling pathway in RCC Gastric Metastasis. This picture highlights the three major signaling pathways, PI3K/AKT, RAS/MAPK, and Wnt, and some regulatory molecules that play a key role in RCC metastasis. The three signaling pathways are central, promoting proliferation, survival, and migration via downstream effectors (e.g., NF-κB, MMP-9, HIF-1α). Natural drugs (e.g., PAC, Tet, Rhein, Fisetin), some proteins (e.g., RASAL2) and miRNAs (miR-134, miR-497) can target these pathways to suppress RCC metastasis. Similarly, some other molecules, such as LINC00460, can target these pathways to promote RCC metastasis. Targeting these pathways with natural drugs or sncRNAs offers a promising prospect for treating gastric metastasis of RCC. [Created in BioRender. Wang, L. (2025) https://BioRender.com/61ypis6 (Agreement number: HQ28PR74FI)].

Metastatic dissemination of RCC to the stomach is primarily driven by the coordinated dysregulation of PI3K/AKT, Ras/MAPK (Mitogen-activated protein kinase), and Wnt/β-catenin signaling pathways (Figure 2). Additionally, other signaling pathways contribute to this process. These pathways promote the metastasis of RCC by regulating EMT, ECM remodeling, and metabolic reprogramming. Complementary cascades further amplify invasive capacity by modulating angiogenesis, ECM stiffening and exosome-mediated intercellular crosstalk, thereby potentiating RCC metastasis to the stomach.

### 6.1 Cell signaling pathways

The metastatic process of RCC cells was found to be driven by the coordinated activation of the PI3K/AKT, Ras/MAPK, and Wnt/β-catenin pathways. Through these pathways, proliferation, survival, and migratory cues were provided, enabling tumor cells to withstand adverse microenvironments and to establish secondary growth at distant sites.

#### 6.1.1 PI3K/AKT

The PI3K/AKT/mTOR signaling pathway is one of the most common tumor-related signaling pathways and also plays a key role in the progression of RCC. Dysregulation of this pathway can enhance cell survival, proliferation, and invasion (Xie et al., 2025). In RCC cells, downregulation of tumor suppressor genes such as PTEN activates mTOR, promoting aberrant cell growth, proliferation, and protein synthesis (Badoiu et al., 2023). Therefore, pharmacological and genetic inhibition of the PI3K/AKT signaling pathway has been demonstrated to attenuate RCC migration and invasion, highlighting its therapeutic relevance, such as PI3K inhibitor treatment reduces RCC progression (Khalid et al., 2023).

Targeted therapies and immunotherapy for RCC have significantly improved the survival rate of patients with advanced RCC, but there are still drug resistance and adverse effects. Natural compounds, including Poria acid (PAC), tetrandrine (Tet), Dendrobine, gallic acid (GA) and Eupatilin, can target the PI3K/AKT signaling pathway (e.g., PAC/Tet blocks NF-κB activation, and Dendrobine downregulates phosphorylated protein, GA enhances autophagy and eupatilin-induced apoptosis), synergistically inhibit the proliferation, invasion and migration of RCC cells (Yang H. et al., 2024; Jia et al., 2025; Chen et al., 2017; Zhang T. et al., 2024; Zhong et al., 2016). In addition to these natural compounds targeting PI3K/AKT, some RNAs have also shown powerful therapeutic effects. The long intergenic noncoding RNA 460 (LINC00460) has been shown to activate the PI3K/AKT cascade, thereby promoting EMT and enhancing tumour cell migration and invasion (Zhou et al., 2022). Conversely, lncRNA P73 antisense RNA 1T (TP73-AS1) can inhibit the expression of KISS1 (KiSS-1 metastasis-suppressor) by interacting with EZH2, which results in PI3K/AKT/mTOR pathway suppression, inhibition of apoptosis, and consequent enhancement of RCC proliferation and invasion (Liu et al., 2018).

HOXA transcript at the distal tip (HOTTIP) is a newly discovered lncRNA, which can not only inhibit autophagy through the PI3K/AKT/Atg13 signaling pathway (Su et al., 2019) and promote RCC cell proliferation, migration, and invasion, but also exert similar effects by upregulating miR-506 (Zhang C. et al., 2024). Unlike lncRNAs, microRNAs (miRNAs) are non-coding RNA. MiR-193a-3p and miR-224 directly inhibit Alpha-2,3-Sialyltransferase IV (ST3GalIV) through the PI3K/AKT pathway to increase cell proliferation and migration (Pan et al., 2018). While miR-613 can directly downregulate AXL levels and block the PI3K/AKT signaling pathway to inhibit the proliferation, invasion, and migration of RCC (Duan et al., 2023).

Centrosomal protein 55 (CEP55) and Annexin A5 promote EMT by activating the PI3K/AKT/mTOR pathway and enhancing the migration and invasion of RCC cells (Chen et al., 2019; Tang et al., 2017). But Transforming growth factor β inducible protein (TGFBI), also known as BIGH3, upregulates the expression of HIF-1α by inhibiting the PI3K/AKT/mTOR signaling pathway, thereby promoting EMT and RCC cell proliferation (Zhan et al., 2024). IRS-2 regulated by TFE3 promotes tumor progression by activating the PI3K/AKT signaling pathway and enhancing the malignant phenotype (Ishiguro and Nakagawa, 2024). Docking protein 1 (DOK1) also promotes ccRCC proliferation and metastasis (Xie et al., 2024a).

Paradoxically, Tumor protein D52 (TPD52) is a member of the TPD52-like protein family, and Glutamate dehydrogenase 1 (GLUD1) is one of the key enzymes in glutamine metabolism. In RCC cells, both inhibit RCC cell proliferation and migration by inhibiting the activation of the PI3K/AKT/mTOR pathway (Wang L. et al., 2022; Zhao et al., 2017), which suggests the spatiotemporal specificity of the PI3K/AKT pathway in metastasis.

#### 6.1.2 Ras/MAPK

The Ras/MAPK pathway has been recognized as a pivotal signaling network that orchestrates tumor–immune crosstalk, drug sensitivity and resistance across diverse malignancies. In RCC, this pathway has been implicated in the control of proliferation, invasion and metastasis; however, its mechanistic underpinnings remain incompletely delineated (Zheng X. et al., 2024).

Nuclear receptor coactivator 7 (NCOA7) inhibits RCC cell proliferation and metastasis by down-regulating this pathway (Guo et al., 2023). And the human circular RNA hsa_circRNA_101705 (circTXNDC11) (Yang et al., 2021), hypoxia-induced TRIB3 (HIF-1α-dependent), and the multi-pathway activator Clusterin (synergistic with PI3K/AKT and VEGF pathways) all work together to drive RCC invasion, metastasis, and angiogenesis by activating the MAPK/ERK axis (Hong et al., 2019; Shi et al., 2013). Previous studies have shown that inhibition of adenosine A2b receptor (A2bR) can reduce the metastasis of melanoma and triple-negative breast cancer. In RCC, blocking A2bR can also inhibit the growth, migration, and invasion (Yi et al., 2020). Furthermore, the VEGF-MAPK/ERK pathway also plays a central role in TKI-resistant RCC. The low expression of miR-549a promotes VEGF secretion by increasing HIF-1α, activates the VEGFR2-ERK2-XPO5 pathway, forms a self-enhancing loop, and drives an increase in vascular permeability and metastasis (Sun et al., 2015).

In addition, some proteins such as micropeptide MIAC, SPARCL1, ING4, DUSPs, RASAL2, some compounds such as Fisetin, Alpha-Mangostin, Rhein, Gαi1, Morusin, and miRNAs such as miR-106b, miR-134, miR-514a-3p, also inhibited the invasion, migration, and proliferation of RCC cells by regulating the MAPK pathway or its related signaling pathways.

The lncRNA-encoded micropeptide MIAC exerts anti-metastatic activity in RCC through a mechanism involving direct binding to AQP2 and subsequent suppression of the EREG/EGFR signaling pathway (Li et al., 2022). Overexpression of Ras protein activator like 2 (RASAL2) upregulates SOX2 expression, inhibits the activity of ERK and p38 MAPK signaling pathways, and inhibits RCC cell invasion (Wang S. et al., 2020).

SPARC-like 1 (SPARCL1), a member of the acidic secretory protein family, inhibited the expression of phosphorylated p38/c-JNK/ERK MAPKs, suggesting that it suppressed RCC cell migration and invasion via the inactivation of p38/JNK/ERK MAPKs (Ye et al., 2017). Inhibitor of growth 4 (ING4) inhibits the expression of dual specificity phosphatases (DUSP4) and activates the p38 MAPK pathway in RCC cells, thereby upregulating the expression of type I IFN-stimulated genes and promoting RCC cell proliferation and metastasis (Tang et al., 2022). CD40/CD40L engagement on RCC cells has been observed to trigger ERK, c-Jun N-terminal kinase (JNK), and p38 MAPK, and to recruit NFAT, thereby driving RCC cell migration (Pontrelli et al., 2021). The G protein alpha inhibitory subunit 1 (Gαi1) can promote RCC cell progression through MAPK-axis activation (Chen et al., 2021). Rhein (4,5-dihydroxyanthraquinone-2-carboxylic acid) and Fisetin inhibit the activation of the MAPK signaling pathway to suppress RCC cell invasion and migration (Ma et al., 2018; Hsieh et al., 2019). Morusin demonstrates cytotoxic effects on RCC cells, inhibiting their proliferation and migration. This cytotoxicity is achieved through the modulation of MAPK signaling pathways, specifically by increasing P-p38 and P-JNK levels while reducing P-ERK levels (Yang et al., 2020). miR-106b can directly target the 3'-UTR of CIC, inhibit the activation of the MAPK pathway, and suppress the proliferation, invasion, and EMT of RCC cells (Miao et al., 2019). The Kirsten rat sarcoma viral oncogene homolog (KRAS) can be targeted and downregulated by miR-134 to inhibit the RAS/MAPK/ERK pathway, thereby inhibiting the proliferation and metastasis of RCC cells (Liu Y. et al., 2015). MiR-514a-3p has been demonstrated to repress the EMT of ccRCC cells by silencing EGFR and its downstream MAPK/ERK signaling pathway (Ke et al., 2017).

It is worth mentioning that in addition to direct regulation, the MAPK pathway can also interact with other signaling pathways to affect the metastasis of RCC to the stomach. For example, CXCR4/CXCL12-mediated activation of the AKT/MAPK axis has been implicated in the promotion of ccRCC metastasis. Following Tpl2 knockdown, CXCR4/CXCL12 downstream signaling is disrupted, AKT and ERK are downregulated, and metastasis is inhibited. Conversely, ERK/JNK MAPK modules are activated by Tpl2, CXCR4 signals are amplified, and ccRCC metastasis is potentiated (Lee et al., 2013). Formin-related protein-1 (FRL1) can promote the MAPK/MMP2 axis to induce EMT, thereby promoting the migration and invasion of ccRCC (Ma et al., 2023). Insulin-like growth factor binding protein 3 (IGFBP3) is a glycoprotein and the most important member of the IGFBP family. Cyclovirobuxine (CVB) can block the IGFBP3- AKT/STAT3/MAPK-Snail pathway. Downregulated Snail expression inhibits the survival, proliferation, apoptosis, angiogenesis, EMT, and migration and invasion of ccRCC cells (Liu et al., 2021).

In summary, the Ras/MAPK pathway plays an important role in the gastric metastasis of RCC, and its regulatory mechanism also involves interactions with other signaling pathways.

#### 6.1.3 Wnt

Activation of Wnt signaling in RCC is associated with glycolytic reprogramming and EMT, processes that are essential for metastatic colonization.

LETM1 (leucine zipper-EF-hand containing transmembrane protein 1), UBE3C, and miR-543 promote the proliferation, migration, and invasiveness of RCC cells by activating the Wnt/β-catenin signaling pathway (Xu et al., 2018; Wen et al., 2015; Chen et al., 2018). Aldolase A (fructose-bisphosphate aldolase A, ALDOA) promotes EMT and enhances the proliferation, migration and invasion abilities of RCC cells by up-regulating the Wnt/β-catenin signaling pathway (Khalid et al., 2023). Linking glycolysis with Wnt activation indicates metabolic dependence in metastasis.

Pygopus 2 (Pygo2) is a novel component of the Wnt signaling pathway, but it does not activate the expression of MMP-9 through the classical Wnt pathway (Liu R. et al., 2015). Kindlin-2 can activate the Wnt signaling pathway to promote the progression of RCC (Li et al., 2017). CXXC4 and IGFBP-4 as endogenous inhibitors may also participate in RCC metastasis through the Wnt/β-catenin signaling pathway, but the specific mechanism still needs to be further understood (Kojima et al., 2009; Ueno et al., 2011).

#### 6.1.4 Other signaling pathways

In addition to the three classical signaling pathways mentioned above, there are some emerging pathways that also have therapeutic significance for RCC metastasis.

STAT (Signal transducer and activator of transcription) is a family of cytoplasmic proteins. STAT3 can be continuously activated by non-receptor tyrosine kinases, such as Janus kinase (JAK), which constitutes the JAK/STAT3 pathway and participates in the progression of RCC (Zhong et al., 2016; Xu et al., 2022). interferon-stimulated gene 15 (ISG15) can promote the proliferation, migration, invasion, apoptosis and cell cycle progression in ccRCC by activating (Xie et al., 2024b). Circ-IP6K2 downregulates the tumor suppressor CAMK2N1 in ccRCC by activating the miR-1292-5p/CAMK2N1 axis, inhibiting the proliferation, migration and invasion of tumor cells *in vitro* (Tang et al., 2024). Inhibitor of nuclear factor kappa B kinase subunit epsilon (IKBKE) upregulates ribonucleotide reductase M2 (RRM2) by activating the RRM2-AKT pathway, thereby promoting the progression of RCC (Liu et al., 2024). The key regulatory molecules in these classical and emerging pathways, as well as their impacts on gastric metastasis of RCC are summarized in Table 1.

**TABLE 1:** Targeted molecules in RCC gastric metastasis signaling pathways.

Molecular name	Type	Target pathway	Effect on RCC metastasis	Ref.
PI3K/Akt/mTOR pathway				
PAC	Natural compound	PI3K/AKT/NF-κB	Suppresses gastric metastasis	Yang et al. (2024a)
Tet	Natural compound	AKT/NF-κB/MMP-9	Suppresses gastric metastasis	Chen et al. (2017)
Dendrobine	Natural compound	PI3K/AKT/Erk	Suppresses gastric metastasis	Jia et al. (2025)
GA	Natural compound	PI3K/AKT/Atg16L1	Selectively suppresses ccRCC metastasis	Zhang et al. (2024b)
Eupatilin	Natural compound	MAPK, PI3K/AKT	Suppresses gastric metastasis	Zhong et al. (2016)
LINC00460	lncRNA	PI3K/AKT	Facilitate gastric metastasis	Zhou et al. (2022)
TP73-AS1	lncRNA	PI3K/AKT/mTOR	Facilitate gastric metastasis	Liu et al. (2018)
HOTTIP	lncRNA	PI3K/AKT/Atg13	Facilitate gastric metastasis	Su et al. (2019), Zhang et al. (2024c)
miR-193a-3p	miRNA	PI3K/AKT	Facilitate gastric metastasis	Pan et al. (2018)
miR-224	miRNA	PI3K/AKT	Facilitate gastric metastasis	Pan et al. (2018)
miR-613	miRNA	PI3K/AKT via AXL	Suppresses gastric metastasis	Duan et al. (2023)
CEP55	Protein	PI3K/AKT/mTOR	Facilitate gastric metastasis	Chen et al. (2019)
TFE3	Transcription factor	PI3K/AKT	Facilitate gastric metastasis	Ishiguro and Nakagawa (2024)
Annexin A5	Protein	PI3K/AKT/mTOR	Facilitate gastric metastasis	Tang et al. (2017)
Ampelopsin	Natural compound	PI3K/AKT	Suppresses gastric metastasis	Zhao et al. (2021)
DOK1	Protein	PI3K/AKT/GSK-3β	Facilitate gastric metastasis	Xie et al. (2024a)
TGFBI	Transcription factor	PI3K/AKT/mTOR	Facilitate gastric metastasis	Zhan et al. (2024)
Ras/MAPK Pathway				
NCOA7	Transcription factor	MAPK/ERK	Suppresses gastric metastasis	Ljungberg et al. (2019)
circTXNDC11	circRNA	MAPK/ERK	Promotes RCC growth and invasion	Escudier et al. (2016)
CLU	Protein	MAPK, PI3K/AKT, VEGF	Promotes RCC growth and invasion	Krishna et al. (2020)
A2bR	Protein	MAPK/JNK	Suppresses gastric metastasis	Cohen et al. (2005)
miR-497	miRNA	MEK/ERK	Suppresses gastric metastasis	Qiu et al. (2024)
micropeptide MIAC	Micropeptide	PI3K/AKT, MAPK	Suppresses gastric metastasis	Carneiro et al. (2024)
SPARCL1	Protein	p38/JNK/ERK MAPKs	Suppresses gastric metastasis	Yamashita et al. (2024)
ING4	Protein	p38 MAPK	Promotes RCC growth and invasion	Namikawa et al. (2012)
RASAL2	Protein	ERK, p38 MAPK	Suppresses gastric metastasis	Bernshteyn et al. (2020)
CD40	Protein	ERK, c-Jun NH(2)-terminal kinase(JNK) and p38 MAPK	Promotes RCC growth and invasion	Zhuang et al. (2019)
Gαi1	Protein	AKT-mTOR, ERK-MAPK	Promotes RCC growth and invasion	Saifi et al. (2023)
Rhein	Natural compound	MAPK/NF-κB	Suppresses gastric metastasis	Mori et al. (2024)
Fisetin	Natural compound	MEK/ERK	Suppresses gastric metastasis	Wang et al. (2020a)
Morusin	Natural compound	MAPK	Suppresses gastric metastasis	Zhang et al. (2019)
Alpha-Mangostin	Natural compound	MEK/ERK/MMP-9	Suppresses RCC migration	Shang et al. (2024)
miR-106b	miRNA	MAPK	Suppresses RCC migration	Kumagai et al. (2022)
miR-134	miRNA	RAS/MAPK/ERK via KRAS	Suppresses proliferation and metastasis	Wang et al. (2022a)
miR-514a-3p	miRNA	MAPK/ERK	Suppresses proliferation and metastasis	Li et al. (2023)
Tpl2	Protein	CXCR4/CXCL12-MAPK	Promotes RCC growth and invasion	Li et al. (2024)
FRL1	Protein	MAPK/MMP2	Promotes RCC growth and invasion	Qin et al. (2021)
CVB	Natural compound	IGFBP3-AKT/STAT3/MAPK-Snail	Suppresses proliferation and metastasis	Piao et al. (2022)
Wnt/β-Catenin Pathway				
LETM1	Protein	Wnt/β-catenin	Drives ccRCC invasion	Xia et al. (2022), Xie et al. (2025), Badoiu et al. (2023)
UBE3C	Enzyme	Wnt/β-catenin	Drives ccRCC invasion	Xia et al. (2022), Xie et al. (2025), Badoiu et al. (2023), Khalid et al. (2023)
ALDOA	Glycolytic enzyme	Wnt/β-catenin/EMT	Drives ccRCC invasion	Khalid et al. (2023)
Other Pathways				
ISG15	Protein	IL-6/JAK2/STAT3	Promotes RCC growth and invasion	Liu et al. (2018)
circ_0000274	circRNA	JAK1/STAT3	Promotes RCC growth and invasion	Zhou et al. (2022)
circ-IP6K2	circRNA	miR-1292-5p/CAMK2N1	Suppresses proliferation and metastasis	Liu et al. (2018)
IKBKE	Enzyme	RRM2-AKT	Promotes RCC growth and invasion	Su et al. (2019)

A2bR, Adenosine A2b receptor; CLU, clusterin; CVB, cyclovirobuxine; DOK1, Docking protein 1; FRL1, Formin-related protein-1; GA, gallic acid; IKBKE, inhibitor of nuclear factor kappa B kinase subunit epsilon; ING4, Inhibitor of growth 4; LETM1, The leucine zipper-EF-hand containing transmembrane protein 1; NCOA7, Nuclear receptor coactivator 7; PAC, poria acid; RASAL2, Ras protein activator like 2; Rhein, 4,5-dihydroxyanthraquinone-2-carboxylic acid; SPARCL1, SPARC-like 1; Tet, Tetrandrine; TGFBI, Transforming growth factor β inducible protein.

#### 6.1.5 Tropism of gastric metastasis in RCC

RCC shows a marked propensity to gastric metastasis, a phenomenon that is difficult to fully explain by the combined dysregulation of the PI3K/AKT, Ras/MAPK, and Wnt/β-catenin pathways (Figure 2). Although these pathways synergistically regulate metastasis-related processes such as EMT, ECM remodeling, and microenvironment adaptation, their effects lack organ specificity and are insufficient to elucidate the strict organ tropism mechanism observed in gastric metastasis. To resolve this contradiction, we propose a three-stage mechanistic paradigm based on technological breakthroughs in spatial multi-omics (genomics, transcriptomics, proteomics, and metabolomics) to systematically explain the organ selectivity of gastric metastasis of RCC.

Stage I, Premetastatic niche formation. The primary tumour secretes HIF-2α-dependent angiopoietin-like 4 (ANGPTL4), which instructs gastric stromal cells to downregulate the mucosal protective peptide trefoil factor 1 (TFF1). Concomitantly, MMP3-driven basement-membrane remodelling and heightened vascular permeability generate a permissive soil. Notably, the acidic gastric lumen (pH 1.5–3.5) establishes a feed-forward loop with tumour-derived hypoxia, thereby consolidating the pre-metastatic niche (Schoenfeld et al., 2023).
Stage II, Organ-selective homing. (i) Chemokine navigation. Gastric CXCL12 expression exceeds that of other metastatic sites by three-fold, creating a steep chemotactic gradient that precisely guides CXCR4-expressing RCC cells (Song et al., 2025). (ii) Metabolic adaptive evolution. The extreme acidity imposes selective pressure that is mitigated via HIF-1α/CAIX signaling, while TRIB3-mediated siderosis is suppressed—enriching for acid-tolerant CA9^high clones (Saliby et al., 2024). (iii) Lactate reprogramming. Spatial metabolomics reveals a lactate micro-gradient within the gastric mucosa that is efficiently scavenged by monocarboxylate transporters (MCTs), conferring a bioenergetic advantage to incoming tumor cells (Gavi et al., 2025; Hu et al., 2024). (iv) Microbial synergy. *Helicobacter pylori* instigates NF-κB activation in gastric epithelia, up-regulating CCL20 and fostering CCR6-mediated RCC tethering—an interaction validated in human gaster-RCC co-cultured organoids (Qu et al., 2024). (v) Extracellular-matrix matching. High COL6A2 expression in RCC cells exhibits structural homology with gastric basement-membrane type IV collagen; integrin signaling (SPP1–CD44 axis), thereby augments cellular anchorage (Yang et al., 2025; Zhang J. et al., 2024).
Stage III, Metastatic ecological niche reconstruction. The acidic milieu upregulates PD-L1, recruiting exhausted tissue-resident memory T (Tem) cells and establishing an immunosuppressive microenvironment (Schoenfeld et al., 2023; Xu et al., 2022). T-cell receptor (TCR) clonotyping traced the public clone CASSLQGADYGYTF across primary and gastric lesions, corroborating organ-specific clonal selection (Xu et al., 2022). Furthermore, *H. pylori* co-culture reproducibly amplified CCL20–CCR6 signaling, phenocopying the critical colonization checkpoint. Collectively, RCC gastric tropism emerges from the multi-dimensional convergence of metabolic fitness, immune evasion, and extracellular-matrix fidelity. Future integration of spatial multi-omics datasets with inter-organ organoid co-culture platforms will be imperative to validate this paradigm and to uncover tractable vulnerabilities within the gastric metastatic cascade.

### 6.2 Angiogenesis

Angiogenesis is a key factor in tumor growth and metastasis, especially play a key role in RCC (Table 3). Tumor cells promote the formation of new blood vessels through various pathways, providing the necessary nutrients and oxygen for the tumor while also promoting the spread and metastasis of tumor cells (Yhee et al., 2012). Neovascularisation is orchestrated through the coordinated activation of the vascular endothelial growth factor (VEGF), platelet-derived growth factor (PDGF) and mammalian target of rapamycin (mTOR) signaling axes, whose induction supplies nutrients and oxygen while facilitating tumor cell dissemination (Négrier and Raymond, 2012). The expression of various target genes of VEGFA and PDGF is upregulated by HIF (Hypoxia-inducible factors), which is a transcription factor that regulates genes related to oxygen sensing, angiogenesis, and cell growth, and also a target gene of the tumor suppressor gene VHL (von Hippel Lindau). VHL is a tumor suppressor gene that encodes pVHL. The main function of pVHL is to target and degrade HIF, especially HIF-1α and HIF-2α, under normal oxygen conditions (Strikic et al., 2023). Although VEGF signaling predominates, PI3K/AKT/mTOR hyper-activation provides an additional route for HIF-1α stabilization (Posadas et al., 2013). VEGF also integrates the activation of the HIF and AKT/mTOR signaling pathways in ccRCC. Polycystin-1 (PC1), a large multi-pass membrane protein with GPCR-like domains, activates the PI3K/AKT/mTOR pathway, upregulates VEGF, and promotes angiogenesis (Gargalionis et al., 2020).

The RUNX family—RUNX1 (Runt-related transcription factor 1), RUNX2 (Runt-related transcription factor 2), and RUNX3 (Runt-related transcription factor 3)—has been implicated in both normal development and oncogenesis, with RUNX3 functioning as a tumor-suppressor gene across multiple malignancies. RUNX3 expression has been shown to downregulate VEGF production and secretion in RCC cells, thereby attenuating cell migration, invasion, and angiogenesis (Chen et al., 2013). Ribosomal S6 protein kinase 4 (RSK4), a component of the MAPK axis, not only directly stimulates VEGF secretion, but also upregulates EPHA2 by phosphorylating RUNX1, further promoting the migration, invasion and angiogenesis of RCC cells (Ma et al., 2025). Mir-125a-3p can downregulate VEGF expression under the activation of PinX1 (PIN2/TRF1-interacting telomerase inhibitor 1), thereby inhibiting RCC angiogenesis and thus inhibiting RCC progression (Hou et al., 2019). CHIP (C-terminal Hsp70-interacting protein) is an E3 ubiquitin ligase that inhibits RCC cell migration, invasion, and angiogenesis by suppressing the VEGF-VEGFR2 pathway (Sun et al., 2015). RASAL2 is a unique RAS GTPase activating protein (RAS-GAP), which can activate GSK3β by reducing Ser9 phosphorylation, thereby reducing VEGFA expression (Hui et al., 2018). F-box only protein 22 (FBXO22), a substrate receptor of the SKP1-Cullin 1-F-box protein (SCF) E3 ubiquitin ligase, has been found to inhibit VEGF-mediated angiogenesis (Guo et al., 2019). Maslinic acid has been observed to inhibit VEGF expression and to inhibit cell proliferation (Thakor et al., 2017). Secreted angiogenic factor that is almost exclusively expressed by endothelial cells and stimulates tumor angiogenesis in a manner similar to VEGF expressed by non-endothelial cell types (such as fibroblasts). miR-126 inhibits the proliferation, migration, and angiogenesis of RCC cells by downregulating EGFL7 expression and inhibiting the downstream ERK/STAT3 signaling pathway. lncRNA HOTAIR, as a competing endogenous RNA (ceRNA), may promote RCC development (Guo et al., 2021). RNA interference directed against VEGF or EGFL7 has been shown to attenuate angiogenesis and tumor growth in RCC (Gu et al., 2015; Xu et al., 2014).

Beyond VEGF, there are also angiogenesis-related genes such as tyrosine-protein kinase receptor-2 (TIE2), angiopoietin 2 (ANG2) and caveolin-1 (CAV1). The oncogenic protein Myoferlin (MYOF) upregulates these genes and promotes RCC migration (Cox et al., 2020). Estrogen receptor β (ERβ) promotes RCC progression by altering angiopoietin (ANGPT-2)/Tie-2 signal-mediated angiogenesis. Therefore, using the anti-estrogen drug ICI182,780 to inhibit ERβ/ANGPT-2/Tie-2 signaling can increase the sensitivity of sunitinib and better inhibit the progression of ccRCC (Gu et al., 2020).

In addition to the regulation of canonical angiogenic genes such as VEGF and TIE2, multiple alternative mechanisms have been identified that govern RCC neovascularization. For example, Branched-chain keto-acid dehydrogenase kinase (BCKDK), the rate-limiting enzyme of branched-chain amino acid (BCAA), the rate-limiting enzyme of branched-chain amino-acid catabolism, has been shown to enhance angiogenesis by up-regulating exosomal miR-125a-5p and concomitantly down-regulating its downstream targets (Yang et al., 2023). Conversely, E74-like transcription factor 5 (ELF5), which belongs to the E26 transformation-specific (ETS) family, stabilizes USP3 through transcriptional activation, significantly weakens angiogenic ability, and acts as a tumor suppressor gene. It is worth mentioning that ELF5 is downregulated in RCC (Ding et al., 2024).

VHL can act as an E3 ubiquitin ligase to ubiquitinate the α subunit of HIF in an oxygen-dependent manner; under normoxic conditions, this binding can degrade the α subunit of HIF. However, under hypoxic conditions, HIF is not degraded, and the HIF signaling cascade promotes angiogenesis. Recently, the role of the VHL-HIF pathway in RCC has been emphasized, and HIF-2α inhibitors have also become a treatment option for patients with metastatic RCC (Roviello et al., 2024). HIF-1α itself behaves as an oncogene that accelerates tumor growth and dissemination by sustaining hypoxia-responsive signaling and up-regulating HIF-2α target genes, including cyclin D1, c-Myc, VEGF-A, EGFR, TGF-α, and GLUT-1 (Mazumder et al., 2023). Gramicidin A is a prototype channel-forming ion carrier that can also inhibit angiogenesis by inhibiting (David et al., 2014).

TKI-resistant RCC cells are highly susceptible to metastasis. They reduce miR-549a through the VEGFR2-ERK-XPO5 pathway, which in turn promotes the expression of HIF-1α, resulting in increased VEGF secretion and further activation of VEGFR2 to form a feedback effect (Xuan et al., 2021). p53 downregulates HIF-1α in RCC, while p53 overexpression reduces VEGF production. The increase of transglutaminase 2 (TGase 2) promotes angiogenesis by inducing p53 degradation, leading to HIF-1α activation. The interaction between HIF-1α and p53 with cofactor p300 is required for stable transcriptional activation. TGase 2-mediated p53 depletion promotes angiogenesis by increasing HIF-1α-p300 binding in RCC (Lee et al., 2020). Polypyrimidine tract-binding protein 1 (PTBP1) is a heterogeneous ribonucleoprotein that can inhibit cell migration, invasion, and angiogenesis *in vitro* and lung metastasis of RCC *in vivo* through (Shan et al., 2018). ELR510444 is a promising new hypoxia-inducible factor inhibitor that reduces the expression of both HIF-1α and HIF-2α, inhibits angiogenesis, and has vascular destructive properties (Carew et al., 2012).

lncRNA-ECVSR can enhance ERβ expression and upregulate HIF-2α to exert its effects. Sunitinib is a first-line drug for the treatment of RCC, and it mainly works by inhibiting tumor angiogenesis. It is worth noting that the lncRNA-ECVSR/ERβ/Hif2-α signaling pathway increased by sunitinib leads to an increase in the cancer stem cell (CSC) phenotype, thereby promoting the formation of vascular mimicry (VM) (He et al., 2022). Migration and invasion inhibitory protein (MIIP) contains 388 amino acids and is considered to be a novel tumor suppressor that can inhibit RCC proliferation and angiogenesis by negatively regulating (Yan et al., 2021).

In summary, angiogenesis in RCC is a complex process involving the interaction of multiple molecules and signaling pathways (Table 2). A deeper understanding of these regulatory mechanisms will help provide new targets and strategies for the treatment of RCC.

**TABLE 2:** Key regulators of angiogenesis in RCC gastric metastasis.

Molecular name	Type	Key pathway/Effector	Effect on RCC metastasis	Ref.
VEGF-related targets				
VEGF-A	Growth factor	VEGFR2/ERK	Promotes RCC growth and invasion	Négrier and Raymond (2012), Strikic et al. (2023)
RUNX3	Transcription factor	VEGF/RASAL2-GSK3β	Inhibits migration and vascularization	Chen et al. (2013)
miR-125a-3p	miRNA	VEGF/STAT3	Suppresses proliferation and metastasis	Hou et al. (2019)
CHIP	Enzyme	VEGF/PI3K/AKT	Suppresses proliferation and metastasis	Sun et al. (2015)
RASAL2	Protein	VEGFA	Suppresses proliferation and metastasis	Hui et al. (2018)
FBXO22	Ubiquitin ligase	SCF-VEGF	Suppresses proliferation and metastasis	Guo et al. (2019)
HIF-Related Targets				
HIF-2α	Transcription factor	VHL-HIF-2α	Promotes metastatic niche formation	Roviello et al. (2024), Mazumder et al. (2023)
Gramicidin A	Ionophore	HIF-1α/TGF-α	Inhibits vascular remodeling	Chen et al. (2021)
TGase 2	enzyme	HIF-1α	Promote angiogenesis	Lee et al. (2020)
PTBP1	Protein	HIF-1α	Suppresses angiogenesis	Shan et al. (2018)
ELR510444	Small molecule	HIF-1α/GLUT-1	Reduces hypoxia adaptation	Carew et al. (2012)
lncRNA-ECVSR	lncRNA	ERβ/Hif2-α	Promote VM	He et al. (2022)
MIIP	amino acid	HIF-2α-CYR61	Inhibit angiogenesis of RCC	Yan et al. (2021)
Other Mechanisms				
ERβ	Protein	ANGPT-2/Tie-2	Suppresses proliferation and metastasis	Gu et al. (2020)
EGFL7	Secreted protein	ERβ/ANGPT-2/Tie-2	Enhances endothelial cell recruitment	Guo et al. (2021), Xu et al. (2014)
BCKDK	Metabolic enzyme	BCAA metabolism	Promotes vascular leakage	Yang et al. (2023)
ELF5	Transcription factor	ETS/USP3	Tumor suppressor	Ding et al. (2024)

CHIP, E3 ligase; ERβ, estrogen receptor β; MIIP, migration and invasion inhibitory protein; PTBP1, Polypyrimidine tract-binding protein 1; TGase, 2, Transglutaminase 2.

### 6.3 Alterations in the extracellular matrix

Upon arrival in the stomach, RCC cells are accompanied by ECM remodeling that both shapes the local microenvironment and fosters tumor adaptation and expansion. Tumor cells can also destroy the ECM by secreting MMPs (Table 3), thereby promoting the infiltration and migration of tumor cells (Mao et al., 2021). MMPs, a family of 24 zinc-dependent endopeptidases whose proteolytic activity is counterbalanced by four tissue inhibitors of metalloproteinases (TIMP-1, TIMP-2, TIMP-3, and TIMP-4). They regulate the proteolytic activity of MMPs by blocking the active sites of MMPs. Both MMPs and TIMPs play an important role in ECM regulation and remodeling. Among them, MMP-9 is the most important and is highly expressed in metastatic RCC (Hashmi et al., 2021).

**TABLE 3:** ECM remodeling and MMP regulation in RCC metastasis.

Molecular name	Type	Mechanism	Effect on RCC metastasis	Ref.
MMP activators				
MMP-9	Protease	Degrades collagen IV and laminin; upregulated by GPER/MAPK/PI3K-AKT	Enhances invasion and migration	Mao et al. (2021), Hashmi et al. (2021), Guan et al. (2018)
FFA4	GPCR	Activates PI3K/AKT/NF-κB to stimulate MMP-9 secretion	Promotes ECM degradation	Karmokar and Moniri (2023)
MMP Inhibitors				
MUC15	Mucin	Suppresses EGFR/PI3K-AKT signaling to reduce MMP-2/9	Inhibits invasion	Yue et al. (2020)
TQ	Natural compound	Downregulates MMP-9 via PI3K/AKT and p38 inhibition	Reduces cell motility	Park et al. (2021)
TPM1	Cytoskeletal protein	Increases E-cadherin and decreases MMP-9	Suppresses EMT	Wang et al. (2019)
TIMPs and Regulators				
Flavonoids	Natural compounds	Upregulate TIMPs and inhibit PI3K/MAPK/STAT3 pathways	Dual anti-angiogenic/anti-invasive	Cayetano-Salazar et al. (2022)
FKBP51	Protein	Link TIMP3 with the Beclin1 complex	Promotes RCC growth and invasion	Mao et al. (2021)
Other components				
DCN	Protein glycoprotein	Increase the expression of P21 and E-cadherin	Suppresses gastric metastasis	Xu et al. (2016)
SMOC2	Protein	Enhancing EMT through the integrin pathway	Promotes gastric metastasis	Feng et al. (2022)
ILK	Protease	Downregulate the EMT-related transcription factors Snail and Zeb1 to regulate the expression of vimentin and E-cadherin	Suppresses gastric metastasis	Han et al. (2015)

DCN, decorin; FKBP51, FK-506-binding protein 51; ILK, Integrin-linked kinase; SMOC2, SPARC-Related Modular Calcium-Binding; TQ, thymoquinone.

Increased expression of MMP-9 promotes RCC cell invasion and metastasis. G protein-coupled estrogen receptor (GPER), a non-genomic signalling mediator, has been shown to upregulate MMP-9 through downstream MAPK and PI3K/AKT. G-1 promotes RCC cell metastasis by activating GPER to enhance PI3K/AKT/MMP-9 signaling pathway (Guan et al., 2018). MUC15, a cell membrane-associated mucin in a variety of epithelial cells, has been demonstrated to inhibit EGFR and PI3K-AKT signaling pathways *in vivo* and *in vitro*, and downregulate MMP2 and MMP9 expression to inhibit invasive behaviors (Yue et al., 2020). Thymoquinone (TQ), a natural compound derived from Nigella sativa seeds, can also downregulate MMP-9 by inhibiting the activation of PI3K/AKT and p38, inhibiting the migration of RCC cells (Park et al., 2021). In addition to affecting tumor cell migration by directly acting on MMP-9, indirect mechanisms have been identified. NF-κB, a regulator of MMP-9 and a key transcription factor in cancer cells, has been implicated in tumor progression by regulating angiogenesis and inhibiting apoptosis, promoting tumor invasion and metastasis. For example, free fatty acid receptor-4 (FFA4), as a G protein-coupled receptor endogenously activated by medium- and long-chain free fatty acids, promotes the migration and invasion of ACHN cells through the PI3K/AKT/NF-κB signaling pathway (Karmokar and Moniri, 2023). Tumor necrosis factor-α (TNF-α) serves as an additional upstream regulator; the RGD (Arg-Gly-Asp)-toxin peptide has been reported to inhibit TNF-α-induced MMP-9 secretion, resulting in suppressed tumor-cell proliferation (Liu et al., 2021). Furthermore, Tropomyosin-1 (TPM1) has been found to upregulate E-cadherin expression while downregulating MMP-9 and VEGF expression, collectively inhibiting tumor metastasis in RCC (Wang et al., 2019). Distinct microRNA networks also converge on MMP-mediated pathways. Whereas miR-106b, miR-134 and miR-514a-3p suppress RCC invasion and migration through MAPK-related signaling, has-miR-15b-5p, has-miR-99b-5p, and has-miR-181a-5p have been demonstrated to interact directly with MMP-2 and MMP-9, thereby inhibiting RCC invasion, migration, and proliferation (Király et al., 2024).

Naturally occurring TIMPs regulate MMP-mediated proteolysis. Four inhibitors, TIMP-1, TIMP-2, TIMP-3, and TIMP-4, are primarily involved in inhibiting MMPs, thereby preventing cell migration, invasion, and angiogenesis (Koochekpour et al., 1999). Consistent with this function, elevated TIMP expression is associated with reduced tumor growth. Plant-derived flavonoids—ubiquitous polyphenols—have been demonstrated to upregulate TIMPs and, simultaneously, to modulate PI3K-AKT, MAPK, NF-κB, signal transducer and activator of transcription 3 (STAT3), and focal adhesion kinase (FAK) signaling cascades that govern cell motility and metastasis (Cayetano-Salazar et al., 2022). FK-506-binding protein 51 (FKBP51) is a high molecular weight protein encoded by the FKBP5 gene, and FKBP51 can promote the autophagic degradation of RCC (Mao et al., 2021).

In addition to MMPs, TIMPs and other important components of the ECM such as IL-2, IFN-α, Decorin (DCN), SMOC2 (SPARC-Related Modular Calcium-Binding), which affect the migration of RCC cells through the cytoplasmic matrix. DCN, a small leucine-rich proteoglycan synthesized by fibroblasts, has been shown to upregulate p21 and E-cadherin, thereby restraining proliferation and migration (Xu et al., 2016). SMOC2 is a matrix cell protein that enhances EMT through the integrin pathway and promotes the growth of RCC cells in the tumor (Feng et al., 2022). High concentrations of IL-2 and IFN-α produced locally at the RCC tumor site directly change tumor characteristics associated with the invasion and metastasis phenotype of RCC (Hathorn et al., 1994). Integrin-linked kinase (ILK) is a serine/threonine kinase that downregulates the EMT-related transcription factors Snail and Zeb1 to regulate the expression of vimentin and E-cadherin to inhibit invasion and metastasis (Han et al., 2015).

In conclusion, RCC cells provide favorable conditions for their adaptation, growth, infiltration and migration in the tumor microenvironment by changing the composition and regulatory mechanism of ECM. This ECM-centred reprogramming constitutes a pivotal mechanism underlying gastric metastasis of RCC.

### 6.4 Exosomes and metastasis

Extracellular vesicles, notably exosomes, have emerged as pivotal mediators of inter-cellular communication within the tumor microenvironment, ferrying DNA, RNA, proteins, and metabolites that dictate recipient-cell fate (Table 4) (Flora et al., 2023; Rui et al., 2023). Communication between cancer cells that express tumor suppressor genes and those that do not is crucial for the distant metastasis of RCC. For example, in a new metastasis model, VHL not only promotes RCC metastasis by regulating VEGF through HIF to affect angiogenesis, but also exosomes produced by VHL (−) RCC cells can induce EMT, migration, invasion and metastasis in VHL (+) RCC cells (Schoenfeld et al., 2023). Macrophage-derived exosome miR-342-3p inhibits NEDD4L, which subsequently inhibits CEP55 ubiquitination and degradation by activating the PI3K/AKT/mTOR signaling pathway, upregulating CEP55 protein expression, and promoting the growth and metastasis of RCC cells (Feng et al., 2021). Similarly, Macrophage-derived exosomes delivering miR-193a-5p promote the progression of RCC by mimicking TIMP2-dependent angiogenesis (Liu et al., 2022).

**TABLE 4:** Exosome-mediated communication in RCC gastric metastasis.

Molecular name	Type	Source	Key pathway/Effector	Effect on RCC metastasis	Ref.
VHL(−) Exosomes	Extracellular vesicles	VHL-deficient RCC cells	HIF-VEGF/EMT	Induces EMT, migration, and metastasis	Flora et al. (2023)
miR-342-3p	miRNA	Macrophage-derived exosomes	PI3K/AKT/mTOR-CEP55	Promotes RCC proliferation and invasion	Feng et al. (2021)
miR-193a-5p	miRNA	Macrophage-derived exosomes	TIMP2/MMP-9	Facilitates angiogenesis and metastasis	Liu et al. (2022)
miR-127-3p	miRNA	RCC cell-derived exosomes	RAB27A/exosome sorting	Accelerates distant metastasis	Song et al. (2024)
piR_004153	piRNA	RCC cell-derived exosomes	FGF2/SLC7A5/WISP1	Enhances MSC migration and tumor support	Bogusławska et al. (2025)
APOC1 (Mechanism 1)	Lipoprotein	Tumor-associated macrophages	CCL5/CCR5	Promotes immune evasion and metastasis	Ren et al. (2022)
APOC1 (Mechanism 2)	Lipoprotein	RCC cells	Wnt3a/β-catenin	Enhances proliferation and migration	Jiang et al. (2021)
APOC1 (Mechanism 3)	Lipoprotein	RCC cell-derived exosomes	STAT3/VEGF	Facilitates vascular invasion	Li et al. (2020)

Tumor cells release extracellular vesicles (EVs) containing several types of molecules, including small noncoding RNAs (sncRNAs) such as miRNAs, piRNAs, or tRNAs (Bogusławska et al., 2025). Within this network, RAB27A—a small GTPase that orchestrates exosome secretion—has been shown to drive the export of miR-127-3p, thereby accelerating RCC metastasis (Song et al., 2024). RAB27B has an oncogenic role in RCC cell lines, but its specific role in exocytosis has not yet been determined (Tsuruda et al., 2020). In hypoxic niches, tumour-associated macrophage-derived exosomes deliver miR-155-5p, which activates the IGF1R/PI3K/AKT cascade and promotes RCC progression (Gu et al., 2021). PiRNAs are sncRNAs with a length of 21–35 nucleotides that regulate gene expression through multiple mechanisms. Mesenchymal stromal cells (MSCs), integral components of the tumor microenvironment, have been reported to respond to the RCC-secreted piRNA piR_004153; this sncRNA alters MSC expression of FGF2, SLC7A5 and WISP1 and enhances MSC migration and survival, providing the first demonstration that cancer-derived piRNAs can augment stromal cell motility (Bogusławska et al., 2025).

Apolipoprotein C1 (APOC1) is the smallest apolipoprotein (6.6 kDa) and is a component of triglyceride-rich lipoproteins and high-density lipoproteins. Macrophages overexpressing APOC1 in RCC promote the metastasis of RCC cells by secreting CCL5 (Ren et al., 2022). APOC1 is upregulated as an oncogene in RCC and promotes the proliferation, migration and invasion of RCC by activating Wnt3a signaling pathway (Jiang et al., 2021). Furthermore, ApoC1 in exosomes is transferred from ccRCC cells to vascular endothelial cells and promotes the metastasis of ccRCC cells by activating STAT3 (Li et al., 2020).

This review introduces the mechanism underlying RCC metastasis to the stomach, integrating PI3K/AKT-driven survival, Ras/MAPK-mediated motility, and Wnt-orchestrated metabolic plasticity with concurrent angiogenic reprogramming, extracellular-matrix remodeling, and exosome-mediated signaling. Although the precise mechanism remains unresolved, the outlined pathway network provides a rational framework for targeted intervention; further investigation is required to clarify these processes.

## 7 Treatment

The treatment strategy for gastric metastasis of RCC should be developed based on the burden of metastasis and the individual situation of the patient. For patients with oligometastasis or isolated gastric metastasis, surgical treatment is an important choice, often using gastrectomy or endoscopic submucosal dissection. As shown in Table 5, gastric metastasis of renal cell carcinoma (RCC) exhibits high heterogeneity in terms of metastatic burden, anatomical location, primary tumor characteristics, and biomarker expression. Patients with oligometastasis (e.g., Cases 3,7) typically present with a single gastric lesion, a longer metastasis-free interval (with time to metastasis [TTM] mostly >24 months), and favorable International Metastatic Renal Cell Carcinoma Database Consortium (IMDC) risk stratification. They are often treated with surgery (gastrectomy or endoscopic submucosal dissection [ESD]) or combined immunotherapy, and some achieve complete response (CR) or long-term stability. In contrast, patients with polymetastasis (e.g., Cases 9, 10, 12) are frequently accompanied by multiple gastric or lung metastases, shorter TTM, and poor IMDC stratification. The efficacy of single targeted therapy (e.g., sunitinib) is limited (with a median overall survival [OS] of approximately 7 months), and disease progression is prone to occur. These data further support that a stratified treatment strategy should be developed based on metastatic burden: patients with oligometastasis can benefit from local treatment (surgery/endoscopy) and may even achieve long-term survival, while patients with polymetastasis require more potent systemic therapy or combined strategies. This is also illustrated in Supplementary Table S1. Case data (Supplementary Table S1) shows (cases 2, 5, 6, 8, 9) that gastrectomy combined with immunotherapy (such as case 2) can achieve CR, while endoscopic treatment alone (case 16) may only achieve disease stability. Similarly, in oligometastatic diseases of other organs (such as the thyroid gland), organ-oriented surgery (such as the thyroid ectomy) has also been shown to improve survival outcomes in selective patients (Aydoğdu et al., 2024). Given that gastric RCC metastasis overlaps with primary gastric cancer, gastrointestinal stromal tumors (GIST), and metastatic melanoma in imaging and endoscopy, establishing a practical diagnostic pathway is crucial (Kim et al., 2022).

**TABLE 5:** Previously published case summary.

Types of metastasis	Case ID	Number of lesions	Anatomic location	IMDC risk group	Primary tumor Size(mm)	Histological subtype	Time to Metastasis(months)	PD-L1/TMB/Biomarkers	OS	PFS	Grade	Ref.
Oligometastasis	1	One polypoid lesion (3 cm) in the stomach, multiple metastases in both lungs	Gastric greater curvature, bilateral lungs	NM	30	ccRCC	252	NM	NM	NM	C	McIlwaine et al. (2022)
	2	One erosive lesion (0.6 cm) in the stomach, one tumor (5 cm) in the right kidney	Anterior wall of gastric body, lower pole of right kidney	Favourable	50	ccRCC	NM	NM	NA	NA	C	Kim et al. (2012)
	3	One metastatic focus in the stomach	Specific location within the stomach not explicitly described	Favourable	30	ccRCC	30	NM	NA	NA	C	Li et al. (2023)
	4	One space-occupying lesion in the stomach, one tumor (8 × 10 cm) in the right kidney	Gastric fundus, mid-upper pole of right kidney	NM	80	ccRCC	13	NM	NM	NM	C	Parmar et al. (2021)
	5	Single gastric lesion (2.5 cm)	Upper gastric body	Favourable	25	ccRCC	72	NM	NA	NA	C	Namikawa et al. (2012)
	6	One gastric polyp; Adrenal/rib/lung metastases	Gastric fundus; Left adrenal gland; Rib; Lung	NM	130	ccRCC	24	IHC: Pax-8+, CK7-, CD68^−^	NM	NM	C	Degerli et al. (2022)
	7	One initially discovered; another one found in the mid greater curvature of the stomach 2 years later	Mid greater curvature of the stomach	Favourable	12	ccRCC	24	NM	NA	NA	C	Kumagai et al. (2022)
Polymetastasis	8	Single gastric lesion (15 mm)	Gastric body	NM	45	ccRCC	72	IHC: CA9+, CD10^+^, CAM5.2+, CK7-	NA	NA	C	Yamashita et al. (2024)
	9	Multiple lung nodules; Multiple gastric submucosal tumors	Bilateral lungs; Gastric body	NM	11	ccRCC	131	NM	NA	NA	B	Mori et al. (2024)
	10	One large mass (8 cm) in the stomach	Gastric cardia, involving most of the gastric fundus, lungs	Poor	37	ccRCC	108	NM	NM	NM	C	Tapasak and McGuirt (2022)
	11	Three mucosal polypoid lesions in the stomach	Gastric fundus and body	Poor	42	NM	96	NM	NM	NM	B	Namikawa et al. (2012)
	12	Multiple lung nodules; Multiple gastric submucosal tumors	Bilateral lungs; Gastric body	Intermediate	40	NM	72	NM	10 months	2 months	C	Saifi et al. (2023)
	13	Three initially discovered (2 pedunculated, 1 protruding); four new polypoid lesions found on follow-up endoscopy 4 months later	Gastric body (upper and middle portions)	Intermediate	70	ccRCC	48	NM	About 7 months	NM	C	Su and Fang (2023)
	14	Three actively bleeding polypoid lesions (diameter 2–3 cm)	Gastric body	NM	35	ccRCC	120	NM	6 months	NM	C	Pezzoli et al. (2007)

ccRCC, clear cell Renal Cell Carcinoma; IHC, immunohistochemistry; IMDC, international metastatic renal cell carcinoma database consortium; NM, not mentioned; NA, not applicable; OS, overall survival; PFS, Progression-Free Survival; TMB, tumor mutational burden; PD-L1, Programmed Death-Ligand 1; CR, complete response; PR, partial response; SD, stable disease; PD, progressive disease; SOL, Space-Occupying Lesion.

The diagnostic pathway should include: (1) When to suspect: For patients with a history of RCC (especially clear cell carcinoma), if gastroscopy reveals hyper-vascular submucosal masses or polyp-like lesions, or if enhanced CT shows a markedly enhanced gastric wall mass, gastric RCC metastasis should be highly suspected. Due to its rarity (0.2%–0.7%) and low detection rate on CT, maintaining high vigilance is essential to avoid misdiagnosis (Magara et al., 2024). (2) First-line examination and biopsy: Endoscopic biopsy is the gold standard for diagnosis. Deep biopsy or endoscopic ultrasound-guided fine needle aspiration (EUS-FNA) is recommended to obtain sufficient tissue and avoid false negatives. Immunohistochemistry (IHC) is central to differential diagnosis, and first-line markers should include PAX8 (highly specific for RCC), CAIX (highly expressed in ccRCC), and CD10. This combination effectively differentiates primary gastric cancer (usually PAX8 negative), GIST (expressing CD117, DOG-1), and melanoma (expressing S100, HMB45) (Gulati et al., 2024). (3) Systemic evaluation: Once pathological diagnosis is confirmed, systemic evaluation must be conducted immediately to ascertain metastatic burden (oligometastasis vs. multiple metastases), which is fundamental for formulating treatment strategies. Enhanced CT (chest, abdomen, pelvis) is preferred, or 18F-FDG PET/CT can be selected depending on the situation (Kim et al., 2022).

The 5-year survival rate of patients with oligometastasis after surgery is 20%–30%, but the surgical effect is limited when multiple metastases are present (such as cases 8 and 10), and systemic treatment is required (Kim et al., 2012; Mori et al., 2024). Treatment options depend on the systemic disease status, patients’ performance status, and severity of symptoms. For patients with isolated gastric metastasis and good performance status, surgical resection can be considered and is associated with prolonged survival in some cases; whereas for those with disseminated disease or poor health, palliative measures such as radiation therapy or embolization may be more suitable for symptom control (Kim et al., 2022). The drug treatment system mainly focuses on targeted therapy and immunotherapy. Among them, the single drug treatment of targeted drugs such as sunitinib and cabozantinib has limited efficacy in treating patients with multiple metastases (median OS of only 7 months in case 15), often due to the development of acquired resistance. Despite the success of VEGF-targeted therapies and mTOR inhibitors in the treatment of advanced RCC, their efficacy is often limited by acquired resistance, which poses a significant challenge in managing advanced diseases such as gastric metastases. The mechanisms of resistance are complex but mainly include the following aspects: Firstly, inhibition of VEGF can lead to the upregulation of other pro-angiogenic factors (such as IL-6, IL-8, Ang-2, FGF, etc.), thereby mediating angiogenesis escape and maintaining tumor blood supply. Moreover, blocking VEGF exacerbates tumor hypoxia, stabilizes HIF-2α, and promotes the expression of its downstream genes (such as VEGF, CXCR4), while recruiting myeloid-derived suppressor cells (MDSCs) and upregulating PD-L1, creating an immunosuppressive microenvironment (Rodrigues et al., 2012; Choueiri et al., 2021). Furthermore, resistant tumors often exhibit downregulation of E-cadherin and upregulation of N-cadherin and Vimentin, accompanied by sarcomatoid differentiation, which enhances metastatic capability (Rodrigues et al., 2012). In addition, cancer-associated fibroblasts (CAFs) and MDSCs support resistant survival by secreting factors such as HGF and SDF-1α (Magara et al., 2024). Notably, the acidic microenvironment, unique extracellular matrix, and metabolic stress in gastric metastases may further exacerbate the aforementioned resistance mechanisms. Among them, monotherapy with targeted agents such as sunitinib or cabozantinib has limited efficacy in treating patients with polymetastases (median OS of only 7 months in Case 15). Therefore, combination strategies are paramount; immunotherapy-based combinations can significantly prolong survival (OS of 28 months in Case 3) (Badoiu et al., 2023; Choueiri et al., 2021). Immune checkpoint inhibitors such as nivolumab and pembrolizumab are often used in combination with targeted drugs or surgery. Case 8 achieved disease stability in combination with ipilimumab, while Case2 achieved CR with postoperative immunotherapy. The prognosis shows significant heterogeneity. Oligometastatic patients can achieve better survival benefits through surgical combined with systemic treatment, while multi metastatic patients mainly rely on systemic treatment (Degerli et al., 2022). However, existing data shows that simple targeted therapy (case 15) or endoscopic therapy is prone to progression. Although stereotactic radiotherapy has been reported to have a local control rate of over 90% for oligometastases, its application in cases of gastric metastasis is relatively rare. Current research suggests that novel combination strategies based on understanding these resistance pathways, such as cabozantinib combined with immunotherapy or other mechanism-based combinations, may improve the prognosis of patients with multiple metastases, indicating that further exploration is needed to optimize treatment plans in the future (Choueiri et al., 2021).

Genomics and transcriptomics technologies have great potential for application in the field of gastric metastasis of RCC (Hu et al., 2024; Clark et al., 2019). Since gastric metastasis of RCC is highly heterogeneous and characterized by delayed recurrence, and the molecular mechanism is unclear, multi-omics technology can analyze the characteristics of metastatic lesions and reveal key signaling pathways, so it can provide a basis for the development of specific biomarkers and dynamic monitoring systems (Jonasch et al., 2021; Yu et al., 2023). In addition, single-cell sequencing technology can explore the laws of tumor cell cloning and assist in early diagnosis and prognosis (Yang G. et al., 2024). In terms of treatment, personalized targeted therapy or immunotherapy strategies based on molecular typing are not yet mature (Pal et al., 2024). However, current research is limited by the small sample size and insufficient mechanism research (Méjean et al., 2018). In the future, it may be necessary to collect multi-center data to build a cross-omics database, which may play an important role in the diagnosis and prognosis of RCC.

A critical deficit in current gastric-cancer oligometastasis research is the absence of a uniform definition: case series indiscriminately label 1–5 lesions as “oligo”, whereas the prospective SINDOL trial demonstrates that only patients with ≤3 metastases derive survival benefit from locoregional therapy (Milano et al., 2021). Based on this, we briefly prioritized the most actionable research gaps in gastric metastasis of RCC. Level 1 is to establish an international, multi-center registry powered to overcome the prevailing sample-size bottleneck and capture real-world heterogeneity. Level 2 is Biomarker-guided local therapy. Embed molecular stratification: tumors co-expressing high CA9 and PI3K activity, for example, should be prioritised for surgery plus targeted agents, ensuring that biology rather than lesion count alone dictates treatment allocation (Jonasch et al., 2021; Yu et al., 2023). While Level 3 is unify definition of oligometastatic disease. Harmonize anatomical criteria (≤3 metastases) with radiomics-derived weighting of lesion location, integrating quantitative imaging features to standardize patient selection across trials and clinical practice (Milano et al., 2021).
