# Supplementary material for: Gastric metastasis of renal cell carcinoma: features, mechanisms, and insights from existing literature
Source: Front Cell Dev Biol. 2025 Oct 15;13:1656858. doi: 10.3389/fcell.2025.1656858 (PMC12572941; doi:10.3389/fcell.2025.1656858)
Supplement: Supplementary file 1 [file Table1.docx]

Supplementary Table 1. Previously published case summary

| **Case**  **Number** | **Report**  **Year** | **Sex** | **Age (years)** | **Country** | **Primary Tumor Size(mm)** | **Histological Subtype** | **Types of Metastasis** | **Time to Metastasis(months)** | **Pre transfer treatment modality** | **Post transfer treatment modality** | **Treatment Response** | **OS** | **PFS** | **Ref.** |
| --- | --- | --- | --- | --- | --- | --- | --- | --- | --- | --- | --- | --- | --- | --- |
| 1 | 2023 | Female | 80 | Canada | 30 | ccRCC | Oligometastasis | 252 | Radical nephrectomy | NM | NM | NM | NM | [36] |
| 2 | 2012 | Male | 79 | Korea | 50 | ccRCC | Oligometastasis | NM | Radical nephrectomy | Endoscopic submucosal dissection | Complete Response(CR) | NA | NA | [11] |
| 3 | 2023 | Male | 65 | Italy | 42 | NM | Oligometastasis | 96 | Right total nephrectomy | Targeted chemotherapy and immunotherapy | NM | NM | NM | [34] |
| 4 | 2021 | Male | 65 | UK | 80 | ccRCC | Oligometastasis | 13 | Right radical nephrectomy combined with gastric SOL resection | NM | NM | NM | NM | [35] |
| 5 | 2014 | Male | 67 | Japan | 40 | NM | Oligometastasis | 72 | Right nephrectomy and chemotherapy treatment | Endoscopic submucosal dissection | Progressive Disease(PD) | 10 months | 2 months | [37] |
| 6 | 2012 | Male | 65 | Japan | 25 | ccRCC | Oligometastasis | 72 | NM | Gastrectomy | CR | NA | NA | [38] |
| 7 | 2022 | Male | 73 | Türkiye | 130 | ccRCC | Oligometastasis | 24 | Radical nephrectomy of the right kidney | First line: Sunitinib | NM | NM | NM | [12] |
| 8 | 2024 | Male | 77 | Japan | 45 | ccRCC | Polymetastasis | 72 | Radical nephrectomy | Gastrectomy | Stable Disease(SD) | NA | NA | [31] |
| 9 | 2024 | Male | 50 | Japan | 11 | ccRCC | Polymetastasis | 131 | Left peritoneal laparoscopic nephrectomy and adrenalectomy | Pyloric gastrecto | CR | NA | NA | [39] |
| 10 | 2012 | Male | 73 | USA | 110 | ccRCC | Polymetastasis | 84 | NM | Gastrectomy | SD | NM | NM | [40] |
| 11 | 2021 | Male | 77 | USA | 37 | ccRCC | Polymetastasis | 108 | Left nephrectomy and chemotherapy treatment | Total gastrectomy combined with Roux-en-Y reconstruction and feeding jejunostomy surgery | SD | NM | NM | [41] |
| 12 | 2024 | Male | 70 | Japan | 12 | ccRCC | Polymetastasis | 24 | NM | Gastrectomy | Partial Respose(PR) | NA | NA | [42] |
| 13 | 2017 | Male | 73 | Japan | 60 | ccRCC | Polymetastasis | 26 | Partial nephrectomy  First Line: temsirolimus | Partial gastrectomy  First Line everolimus: | PR | About 4 months | NM | [43] |
| 14 | 2014 | Male | 64 | Japan | 30 | ccRCC | Polymetastasis | 30 | Pylorus preserving pancreaticoduodenectomy(PPPD) | Endoscopic submucosal dissection | CR | NA | NA | [44] |
| 15 | 2020 | Male | 65 | China | 70 | ccRCC | Polymetastasis | 48 | Right radical nephrectomy | First Line: Sunitinib | PD | About 7 months | NM | [45] |
| 16 | 2011 | Female | 70 | NM | 65 | ccRCC | Polymetastasis | 11 | NM | Endoscopic submucosal dissection | SD | 3 months | 1 month | [46] |
| 17 | 2007 | Male | 78 | USA | 35 | ccRCC | Polymetastasis | 120 | Radical nephrectomy | Gastrectomy | PR | 6 months | NM | [47] |
| 18 | 2012 | Male | 45 | NM | 50 | NM | Polymetastasis | 120 | nephrectomy | First line: Sunitinib | PD | 4 months | NM | [48] |

Note: ccRCC, clear cell Renal Cell Carcinoma; CR, Complete Response; PR, Partial Response; SD, Stable Disease; PD, Progressive Disease; NM, Not Mentioned; NA, Not Applicable; OS, Overall Survival; PFS, Progression-Free Survival; SOL, Space-Occupying Lesion; PPPD, Pylorus Preserving Pancreaticoduodenectomy.
